# Perioperative Management of Decompensated Peripartum Cardiomyopathy With Severe Left Ventricular Dysfunction: A Case Report

**DOI:** 10.7759/cureus.118447

**Published:** 2026-10-23

**Authors:** Renard Ramkeesoon, Amanda C Sang, Keevan Singh, Candice Ramdin

**Affiliations:** 1 Anaesthesia and Intensive Care Unit, San Fernando General Hospital, San Fernando, TTO; 2 Anaesthesia and Intensive Care Unit, Department of Clinical Surgical Sciences, University of the West Indies, San Fernando, TTO

**Keywords:** cardiomyopathy, decompensated heart failure, perioperative, peripartum cardiomyopathy, pregnancy

## Abstract

Peripartum cardiomyopathy (PPCM) is a potentially life-threatening cause of heart failure that may be difficult to distinguish from the physiological symptoms of pregnancy. We report the case of a 42-year-old woman at 34 weeks of gestation who presented with progressive dyspnea and was found to have severe global left ventricular systolic dysfunction with an ejection fraction of approximately 20%. Despite initial improvement with diuresis, she developed recurrent acute decompensated heart failure requiring intensive care unit readmission. Point-of-care ultrasonography (POCUS) demonstrated pulmonary congestion and reduced forward flow, with a left ventricular outflow tract velocity-time integral (LVOT VTI) of 10 cm. Following hemodynamic optimization with diuresis and inotropic support, cesarean delivery was performed under general anesthesia with invasive monitoring and titrated vasoactive support. Postoperatively, LVOT VTI increased to 15 cm with corresponding clinical improvement. This case highlights the value of serial hemodynamic assessment, including POCUS, and multidisciplinary perioperative management in severe PPCM with recurrent decompensation.

## Introduction

Peripartum cardiomyopathy (PPCM) is a potentially life-threatening form of heart failure characterized by left ventricular systolic dysfunction, with a left ventricular ejection fraction (LVEF) below 45%, occurring predominantly during the peripartum period or in the months following delivery, in the absence of another identifiable cause of heart failure [1-3]. It remains a diagnosis of exclusion and may present with manifestations ranging from relatively subtle symptoms to severe acute heart failure or cardiogenic shock.

The incidence of PPCM varies considerably across geographical regions and populations, suggesting contributions from genetic, environmental, socioeconomic, and obstetric factors [1-3]. Established risk factors include hypertensive disorders of pregnancy, multiparity, multiple gestation, diabetes mellitus, obesity, and advanced maternal age [1-3]. Contemporary pregnancy patterns, including increasing maternal age and a greater burden of cardiovascular risk factors, make continued clinical awareness particularly important.

Diagnosis may be challenging because common manifestations of PPCM, including dyspnea, orthopnea, fatigue, and peripheral edema, overlap with physiological symptoms of late pregnancy. Echocardiography is therefore central to diagnosis, assessment of ventricular function, and exclusion of alternative structural cardiac pathology. Biomarkers such as B-type natriuretic peptide (BNP) and N-terminal pro-B-type natriuretic peptide (NT-proBNP) may provide additional diagnostic and prognostic information where available [1-3].

Management of severe PPCM during pregnancy presents additional challenges because maternal hemodynamic optimization must be balanced against fetal wellbeing, timing of delivery, and the hemodynamic consequences of anesthesia and the postpartum period. Multidisciplinary management involving cardiology, obstetrics, anesthesia, and critical care is therefore essential. We report the perioperative management of a woman with severe PPCM and an LVEF of approximately 20% who experienced recurrent acute decompensated heart failure before undergoing preterm cesarean delivery, with serial point-of-care ultrasonography (POCUS) incorporated into hemodynamic assessment. Left ventricular outflow tract velocity-time integral (LVOT VTI) is a non-invasive echocardiographic measurement used to estimation of stroke volume and, by extension, cardiac output. Serial measurements may therefore complement clinical assessment in risk stratification and monitoring of treatment response.

## Case presentation

A 42-year-old woman at 34 weeks of gestation with chronic hypertension treated with methyldopa and type 2 diabetes mellitus treated with metformin presented with progressive dyspnea, bilateral lower-limb edema, and a three-day history of productive cough with clear sputum. In the clinic, her blood pressure was 150/100 mmHg and heart rate was 116 beats/minute. She denied headache, visual disturbance, or epigastric pain, and urine dipstick showed no qualitative proteinuria. Her initial bloodwork was performed.

She was admitted to the antenatal ward for blood pressure control and further investigations. Following admission, she developed worsening dyspnea with oxygen saturations of 92% to 95% on room air, chest pain, and tachycardia. Electrocardiography demonstrated sinus tachycardia. Computed tomography pulmonary angiography excluded pulmonary embolism but demonstrated bilateral pleural effusions. Bedside transthoracic echocardiography demonstrated severe global left ventricular hypokinesia with an estimated ejection fraction of 20%, no significant aortic or mitral valve abnormality, and qualitatively preserved right ventricular function. BNP and NT-proBNP assays were unavailable. A diagnosis of PPCM with acute heart failure was made, and she was transferred to the intensive care unit (ICU) for intravenous diuresis and medical management. Laboratory values are presented in Table 1.

**Table 1:** Laboratory values.

Parameter	Value	Reference range
Hemoglobin	11.6 g/dL	11.0–14 g/dL
Platelet count	215 × 10⁹/L	159–388 × 10⁹/L
Creatinine	0.7 mg/dL	0.6–1.3 mg/dL

It was documented by the managing obstetric team that the fetus was in a transverse lie and had intrauterine growth restriction, with an estimated fetal weight of 1.69 kg; cardiotocography remained reassuring. Given the improvement in maternal condition and reassuring fetal status, delivery was deferred to allow further fetal maturation, and antenatal dexamethasone was administered at 12 mg intramuscular injection for one day. After five days, she was transferred from the ICU to a high-acuity ward while awaiting planned cesarean delivery.

Forty-eight hours later, she developed recurrent acute respiratory deterioration despite ongoing diuretic therapy. She was sitting upright, diaphoretic, unable to speak in full sentences, and had cool extremities with a capillary refill time greater than three seconds. SpO₂ was 90-92% on supplemental oxygen, blood pressure was 105/65 mmHg (mean arterial pressure: 78 mmHg), and heart rate was 110 beats/minute. Given the presence of significant heart failure symptoms at rest, her functional status at the time of decompensation was consistent with New York Heart Association (NYHA) [4] functional class IV. POCUS demonstrated bilateral B-lines, a dilated inferior vena cava with <50% collapsibility, and an LVOT VTI of 10 cm. Serum creatinine increased from 0.7 to 1.0 mg/dL within 24 hours, meeting the creatinine criterion for Kidney Disease: Improving Global Outcomes (KDIGO) [5] Stage 1 acute kidney injury despite preserved urine output. Serum lactate was unavailable. These findings were consistent with recurrent congestion with evidence of impaired peripheral perfusion and reduced forward flow.

She was readmitted to the ICU, where dobutamine was commenced at 5 μg/kg/minute and intravenous diuresis was continued. Her respiratory status subsequently improved, with oxygen requirements decreasing to 2 L/minute via a nasal cannula. Initiation of dobutamine also showed an improvement in urine output of approximately 30 mL/hour. Given recurrent decompensated heart failure despite initial medical management, the multidisciplinary team elected to expedite delivery.

At 35 weeks and 4 days of gestation, she underwent cesarean delivery under general anesthesia. This approach was selected in view of her recent cardiopulmonary decompensation, severe left ventricular systolic dysfunction, reduced LVOT VTI, and ongoing inotropic requirement. Dobutamine was continued perioperatively, and invasive arterial pressure monitoring was used. Following preoxygenation, a modified rapid-sequence induction was performed using fentanyl 2 μg/kg, propofol 1 mg/kg, and suxamethonium 1.5 mg/kg. The trachea was secured using video laryngoscopy. Norepinephrine was commenced following induction and titrated between 0.1 and 0.2 μg/kg/minute to maintain arterial blood pressure close to the patient’s pre-induction baseline. A restrictive fluid strategy was employed, with no intraoperative crystalloid administered.

Following induction, she developed atrial fibrillation with rapid ventricular response at approximately 150 beats/minute without associated hemodynamic deterioration. Intravenous amiodarone 150 mg restored sinus tachycardia. A live male neonate weighing 1.7 kg was delivered with an Apgar score of 9 and 10 [6] at one and five minutes, respectively. Oxytocin was administered cautiously as a 3-unit slow intravenous dose followed by an infusion of 10 units/hour. Estimated blood loss was approximately 200 mL.

She was extubated at the completion of surgery and transferred to the ICU. Dobutamine and norepinephrine were subsequently discontinued. Serial POCUS demonstrated an increase in LVOT VTI from 10 to 15 cm, accompanied by resolution of respiratory distress. Serum creatinine returned to baseline at 0.7 mg/dL before discharge. She was transferred to the ward and discharged home 48 hours later, with cardiology follow-up arranged.

## Discussion

PPCM is a potentially life-threatening cause of heart failure in pregnancy [1]. Diagnosis can be challenging because dyspnea, fatigue, and peripheral edema may initially be attributed to physiological changes of pregnancy [1]. In this patient, advanced maternal age and chronic hypertension were recognized risk factors, while the development of progressive respiratory symptoms and severe global left ventricular systolic dysfunction with an LVEF of approximately 20% prompted consideration of PPCM. Pulmonary embolism was excluded by computed tomography pulmonary angiography, and no major aortic or mitral valvular abnormality was identified. BNP and NT-proBNP were unavailable, making clinical assessment and echocardiography particularly important.

Clinical profiling of acute heart failure according to congestion and perfusion provides a practical bedside framework for hemodynamic assessment. The 2016 European Society of Cardiology heart failure guidelines classify patients according to the presence or absence of congestion (“wet” or “dry”) and peripheral hypoperfusion (“cold” or “warm”), resulting in the following four clinical profiles: warm-dry, warm-wet, cold-dry, and wet-cold [7]. In this patient, recurrent respiratory distress, hypoxemia, bilateral B-lines, and a dilated inferior vena cava supported a “wet” profile. Concurrent cool extremities, capillary refill time greater than three seconds, Stage 1 acute kidney injury, and an LVOT VTI of 10 cm supported impaired peripheral perfusion and reduced forward flow, consistent with a wet-cold phenotype.

Recognition of this phenotype influenced subsequent management. Intravenous diuresis was continued to address pulmonary congestion, while dobutamine was introduced in the setting of clinical hypoperfusion and reduced forward flow. Inotropic therapy is not routinely indicated in PPCM but may be required in selected patients with significant hemodynamic compromise [1,2]. Bedside POCUS complemented serial clinical assessment when natriuretic peptides and serum lactate were unavailable. LVOT VTI provided a reproducible marker of changes in forward stroke distance rather than a direct measurement of cardiac output and, when used in conjunction with blood pressure and lactate levels, can guide shock diagnosis [8]. Following treatment and delivery, VTI increased from 10 to 15 cm, respiratory distress resolved, vasoactive support was withdrawn, and serum creatinine returned to 0.7 mg/dL. These parallel changes supported improvement in congestion, perfusion, and forward flow, although a causal relationship between the POCUS findings and clinical outcome cannot be inferred.

Timing of delivery required repeated assessment of maternal and fetal risk. Following the initial response to treatment and reassuring fetal surveillance, delivery was deferred to permit further fetal maturation and administration of antenatal corticosteroids. Recurrent decompensation at 35 weeks and 4 days altered this balance and prompted expedited delivery. Current guidance emphasizes multidisciplinary, individualized delivery planning in women with significant cardiovascular disease, with cesarean delivery appropriate when acute heart failure or obstetric indications make continued pregnancy or labor undesirable [1-3,9]. The anesthetic technique should similarly be individualized [8]. In this patient, recent cardiopulmonary decompensation, LVEF of approximately 20%, reduced LVOT VTI, and ongoing dobutamine requirement led to selection of general anesthesia with invasive arterial monitoring. Continued inotropic support, titrated norepinephrine, and avoidance of unnecessary intravenous fluid were used to preserve perfusion pressure while minimizing further congestion.

The perioperative period remained high risk because severe PPCM is associated with arrhythmias and vulnerability to rapid hemodynamic change [1,3]. A transient tachyarrhythmia consistent with atrial fibrillation with rapid ventricular response occurred following induction and was treated with intravenous amiodarone without associated cardiovascular deterioration. Oxytocin was administered slowly and followed by an infusion to minimize abrupt hemodynamic effects. Postoperative ICU surveillance permitted continued assessment during the early postpartum period, when rapid changes in preload may be poorly tolerated in severe left ventricular dysfunction.

This case therefore illustrates the value of integrating bedside perfusion findings, renal function, and serial POCUS with conventional hemodynamic monitoring to recognize and manage a wet-cold phenotype of acute decompensated heart failure in severe PPCM.

## Conclusions

Severe PPCM may deteriorate rapidly despite initial clinical improvement. This case highlights the importance of recognizing a wet-cold phenotype through the integration of clinical assessment, renal function, and POCUS, with serial LVOT VTI providing an adjunctive marker of changes in forward flow. Multidisciplinary, individualized management remains essential to guide hemodynamic optimization and timing of delivery.
